# Long-Term Interplay Between SARS-CoV-2 and Renal Impairment

**DOI:** 10.7759/cureus.66553

**Published:** 2024-08-10

**Authors:** Ileana Adela Vacaroiu, Larisa Florina Șerban-Feier, Dragos Eugen Georgescu, Daniela- Gabriela Balan, Mircea Ovidiu Denis Lupușoru, Elena Cuiban, Andrada Doina Mihai, Andra-Elena Balcangiu-Stroescu

**Affiliations:** 1 Department of Nephrology, Faculty of Medicine, Carol Davila University of Medicine and Pharmacy, Bucharest, ROU; 2 Department of Nephrology, "Sf. Ioan" Clinical Emergency Hospital, Bucharest, ROU; 3 Department of General Surgery, Carol Davila University of Medicine and Pharmacy, Bucharest, ROU; 4 Department of General Surgery, Dr. I. Cantacuzino Clinical Hospital, Bucharest, ROU; 5 Department of Physiology, Faculty of Dental Medicine, Carol Davila University of Medicine and Pharmacy, Bucharest, ROU; 6 Department of Diabetes, Nutrition and Metabolic Diseases, Carol Davila University of Medicine and Pharmacy, Bucharest, ROU

**Keywords:** coronavirus disease, relationship, infectious complications, mortality, chronic kidney disease (ckd)

## Abstract

Introduction

The SARS-CoV-2 virus causes the highly contagious coronavirus disease 2019 (COVID-19), which most commonly manifests as severe acute respiratory syndrome. The virus is part of the Coronaroviridae family, a group of viruses that can cause various diseases, such as the common cold, severe acute respiratory syndrome (SARS), and Middle East respiratory syndrome (MERS). The World Health Organization (WHO) declared the outbreak of COVID-19 as a pandemic on March 11, 2020. On February 26, 2020, Romania confirmed the first case of COVID-19, initiating a series of challenges that negatively impacted the lives of thousands of people. The COVID-19 pandemic has had a disproportionate effect on patients at risk of kidney damage. Patients with chronic kidney disease (CKD) are at high risk of SARS-CoV-2 infection and mortality associated with COVID-19. CKD is associated with pronounced immunodeficiency and represents a risk factor for contracting the infection, but also increases the risk of hospitalization, oxygen therapy, and prolonged treatments.

The evidence regarding the management of patients with CKD undergoing renal replacement therapy (RRT) infected with SARS-CoV-2 is still misleading. While these are high-risk patients due to the presence of multiple comorbidities, especially cardiovascular, e.g., hypertension, left ventricular hypertrophy, but also diabetes, the question remains whether RRT itself is associated with a worse prognosis in patients infected with SARS-CoV-2, although infections generally induce severe complications in patients with CKD and RRT.

Methods

This retrospective study aims to analyze the evolution of COVID-19 disease in patients with CKD, focusing on the association with some common comorbidities such as ischemic coronary disease (ICD), obesity, and diabetes. The study included 72 hemodialyzed patients; they were hospitalized between November 2020 and February 2021 at "Sf. Ioan" Clinical Emergency Hospital, Nephrology and Dialysis Clinic; peritoneal dialysis patients were excluded.

Results

Older age was found to be an important risk factor for death in hemodialyzed patients admitted with COVID-19 infection. Obese patients were found to be at greater risk of mortality.

Discussion

This study showed that there is a complex relationship between COVID-19 infection and increased mortality in patients with CKD associating ischemic coronary disease, obesity, and diabetes.

## Introduction

The coronavirus disease 2019 (COVID-19) pandemic has represented a cumulative risk factor for increasing mortality and morbidity globally, particularly among patients with pre-existing comorbidities. By far, patients with chronic kidney disease (CKD) are noted in the medical literature to be significantly affected [1,2], both because of the generally advanced age and because of the pathophysiology and clinical picture of CKD [3]. Many available studies in the current literature come with clear evidence that the risk of mortality is increased in patients with COVID-19 and CKD when compared to those without CKD. Similar to mortality, CKD patients are at increased risk of hospitalization, notably in advanced stages of CKD. At the start of the pandemic, approximately 50% of CKD patients on hemodialysis diagnosed with COVID-19 required hospitalization, and mortality rates ranged from 20 to 30%. Patients who performed hemodialysis in a chronic facility were three to four times more likely to be hospitalized with COVID-19 when compared to peritoneal dialysis patients, who weren’t exposed to interactions with medical personnel and other patients. The male gender is associated with a higher death risk as compared to the female gender [4].

Obesity, diabetes, severe forms of asthma, other chronic respiratory diseases, chronic heart diseases, liver diseases, a history of stroke or dementia, some autoimmune diseases, and immunodeficiency were among the most common comorbidities that proved to have a negative effect on survival [4].

Decreased renal function, considered both as any decrease in estimated glomerular filtration rate (eGFR) and as requiring renal replacement therapy, was associated in both cases with a higher risk of death, the association being stronger with lower eGFR [4].

There are a multitude of heterogeneous studies on paraclinical prognostic markers, both in terms of methodology and definition of severe form [5]. Some studies considered the prognostic ability of biomarkers for patient admission, others for intensive care admission or need for mechanical ventilation, and others for death.

Possible markers with great potential to predict severe forms of COVID-19 disease in patients with acute or chronic kidney disease are C-reactive protein, procalcitonin, interleukin-6, prothrombin, D-dimers, and serum concentration of platelets [5,6]. Troponin I and serum creatinine have proved to be the most useful when regarding the involvement of the heart and kidneys [5].

An international multicenter study, from 2020, conducted on a group of 4716 CKD patients showed an incidence of COVID-19 in CKD patients of 4.09% [7]. The incidence of COVID-19 in CKD patients is approximately 10 times higher than that in the general population [7]. The same study shows that the incidence of COVID-19 was higher in older CKD patients, associating cardiovascular, pulmonary, or oncological comorbidities, the severity increasing exponentially with the decrease of renal function [7].

Data from the European Renal Association-European Dialysis and Transplant Association (ERA-EDTA) registry on seven European countries, including Romania, showed a mortality of 21.2% [8], of which 20.0% was attributable to the infection. Patients requiring renal replacement therapy in Romania had an unadjusted mortality of 8.5% at 28 days [8]. A Romanian retrospective study has analyzed the mortality of patients on maintenance hemodialysis diagnosed with COVID-19 and demonstrated that 19% of the patients died during hospitalization for COVID-19, and hypoalbuminemia and anemia were associated with high mortality [9]. Advanced CKD stages are strongly associated with increased mortality in patients with COVID-19 disease [10]. Some hypotheses were also studied, like increased cell death in COVID-19 infection and overlapping programmed cell death in CKD [11-13]. Unconcerned with the CKD etiology, the higher risk of mortality and morbidity remains in patients who associate a great burden of cardiovascular comorbidities [14,15]. The aim of this study is to highlight the interaction between COVID-19 and CKD patients, associating some of the most common comorbidities of the CKD patient population: ischemic coronary heart disease, obesity, and diabetes, and to analyze the risk factors for mortality in hemodialyzed patients.

## Materials and methods

The present study is an observational, retrospective, and unicentric study. A group of 72 patients were included; they were hospitalized between November 2020 and February 2021 at "Sf. Ioan" Clinical Emergency Hospital, Nephrology and Dialysis Department.

The General Data Protection Regulation (GDPR), applied in the European Union since May 25, 2018, is respected. The personal identification data of the patients were excluded from the database used for the analysis.

Inclusion criteria

Patients aged > 18 or 18 years; end-stage CKD with renal replacement therapy through hemodialysis; and patients diagnosed with COVID-19 infection by reverse transcription polymerase chain reaction (RT-PCR).

Exclusion criteria

Patients under 18 years of age and on peritoneal dialysis are excluded.

Data collected

Demographic characteristics such as gender and age of patients; pathological antecedents such as the etiology of CKD and the presence of some comorbidities like ischemic coronary disease, obesity, diabetes, hypertension, neoplasms, and lung diseases other than COVID-19; paraclinical examinations at the beginning of hospitalization - serum concentrations of hemoglobin, leukocytes, lymphocytes, albumin, ferritin, interleukin-6 (IL-6), and D-dimers; the evolution of the infection and its complications: if the patients presented infectious complications of any kind (including sepsis), if they developed other vascular acute complications like thrombosis, hemorrhages, and of course death. For statistical analysis, to assess the correlation between two categorical variables, the Fisher's exact test was used. To assess the correlation between a categorical variable and a scalar variable, either the student t-test (in the case of a normal distribution of the scalar variable) or the Mann-Whitney U test (in the case of a non-normal distribution of the scalar variable) are used. The Shapiro-Wilk test is used to determine the nature of the distribution of scalar variables. The p<0.05 threshold is considered statistically significant. Descriptive and analytical statistical analysis are performed using the IBM SPSS Statistics for Windows, Version 25 (Released 2017; IBM Corp., Armonk, New York, United States).

## Results

Ischemic coronary disease

This subgroup includes 31 hemodialyzed patients with ischemic coronary heart disease, hospitalized for COVID-19. Their characteristics are shown in Table 1. We did not find a statistically significant correlation (p=0.704) between gender and the rate of mortality in this subgroup (Table 1). Considering age as a scalar variable, we tested whether the patients who died were older and found a statistically significant correlation (p=0.005) between older age and risk of death. Considering age as a dichotomous categorical variable (<65 years or ≥65 years), we tested whether patients who died were older and found a statistically significant correlation (p=0.009) between older age and death (Table 1). In this subgroup, statistically significant associations with mortality are found for ferritin (p=0.003) (Table 1). Also, we found statistically significant associations with mortality and infectious complications of any kind (p=0.023) (Table 1).

**Table 1:** Characteristic of patients with ischemic heart disease * *Fisher’s exact test; **Shapiro-Wilk test; ***student t-test; ****Mann-Whitney U test

Parameter (N=31)	Value	Survivors	Non-survivors	(p<0.05)*,**,***,****
Gender (Nr. (%))	10 (32.3%) F, 21 (67.7%) M	4 (26.7%) F, 11 (73.3%) M	6 (37.5%) F, 10 (62.5%) M	0.704*
Age (MV ± SD) (years)	68.77 ± 10.862 (p=0.346*)	63.40 ± 8.467 (p=0.817**)	73.81 ± 10.635 (p=0.258**)	0.005***
Age group (Nr. (%))				
<41	0 (0%)	0 (0%)	0 (0%)	0.020*
41-65	11 (35.4%)	9 (60.0%)	2 (12.5%)	-
66-80	14 (45.2%)	5 (33.3%)	9 (56.2%)	-
>80	6 (19.4%)	1 (6.7%)	5 (31.3%)	-
Obesity (Nr. (%)) (N=24)	12 (50.0%) - 12 (50.0%) +	7 (53.8%) - 6 (46.2%) +	5 (45.5%) - 6 (54.5%) +	1.000*
Hypertension (Nr. (%))	1 (3.2%) - 30 (96.8%) +	0 (0%) - 15 (100%) +	1 (6.2%) - 15 (93.8%) +	1.000*
Atherosclerosis (Nr. (%))	11 (35.5%) - 20 (64.5%) +	6 (40.0%) - 9 (60.0%) +	5 (31.2%) - 11 (68.8%) +	0.716*
Neoplasia (Nr. (%))	27 (87.1%) - 4 (12.9%) +	14 (93.3%) - 1 (6.7%) +	13 (81.2%) - 3 (18.8%) +	0.600*
Pulmonar disease (Nr. (%))	25 (80.6%) - 6 (19.4%) +	13 (86.7%) - 2 (13.3%) +	12 (75.0%) + 4 (25.0%) +	0.654*
Diabetes (Nr. (%))	18 (58.1%) - 13 (41.9%) +	10 (66.7%) - 5 (33.3%) +	8 (50.0%) - 8 (50.0%) +	0.473 *
Chronic kidney disease etiology (Nr. (%)) (N=25)				
Ischemic nephropathy	16 (64.0%) - 9 (36.0%) +	6 (42.9%)	3 (27.3%)	0.116*
Diabetic nephropathy	17 (68.0%) - 8 (32.0%) +	3 (21.4%)	5 (45.4%)	-
Tubulointerstitial nephropathy	21 (84.0%) - 4 (16.0%) +	4 (28.6%)	0 (0%)	-
Multiple myeloma	23 (92.0%) - 2 (8.0%) +	0 (0%)	2 (18.2%)	-
Other glomerular causes	23 (92.0%) - 2 (8.0%) +	1 (7.1%)	1 (9.1%)	-
Oxigenotherapy (Nr. (%))	19 (61.3%) - 12 (38.7%) +			
Infections (Nr. (%))	20 (64.5%) - 11 (35.5%) +	13 (86.7%) - 2 (13.3%) +	7 (43.8%) - 9 (56.3%) +	0.023*
Hemorrhagic adverse events (Nr. (%))	24 (77.4%) - 7 (22.6%) +	12 (80.0%) - 3 (20.0%) +	12 (75.0%) - 4 (25.0%) +	1.000*
Thrombosis (Nr. (%))	30 (96.8%) - 1 (3.2%) +	15 (100%) - 0 (0.0%) +	15 (93.8%) - 1 (6.3%) +	1.000*
Death (Nr. (%))	15 (48.4%) - 16 (51.6%) +			
Hospitalization lenght (MV ± SD) (days)	15.16 ± 6.229 (p=0.712*)	16.67 ± 5.888 (p=0.090**)	13.75 ± 6.393 (p=0.606**)	0.198***
Lymphocytes (Nr. / μL)	640.0 (470.0-1100.0) (p=0.001*)	650.0 (420.0 - 1260.0) (p=0.131**)	610.0 (515.0 – 860.0) (p<0.001**)	0.812****
Lymphopeniae (Nr. (%))	8 (25.8%) - 23 (74.2%) +	5 (33.3%) - 10 (66.7%) +	3 (18.8%) - 13 (81.2%) +	0.433*
Hemoglobin (g/dL)	10.40 ± 2.025 (p=0.137*)	10.08 ± 1.783 (p=0.101**)	10.70 ± 2.244 (p=0.181**)	0.403***
Albumin (g/dL) (N=28)	3.6357 ± 0.45085 (p=0.945*)	3.7513 ± 0.42470 (p=0.117**)	3.5023 ± 0.45926 (p=0.119**)	0.148***
Ferritin(ng/mL) (N=24)	1789.0 (620.08 - 2663.0) (p<0.001*)	661.45 (309.33 - 1874.50) (p<0.001**)	2107.50 (1652.50 – 3659.75) (p<0.001**)	0.009****
Interleukin – 6 (pg/mL) (N=16)	66.130 (25.0825-103.4525) (p<0.001*)	54.36 (16.300 - 72.905) (p=0.813**)	91.31 (41.280 – 821.40) (p=0.017**)	0.064****
C-reactive protein (mg/dL)	77.50 (29.540-150.110) (p<0.001*)	48.32 (29.54 - 117.46) (p=0.072**)	101.67 (28.4725 - 179.12) (p=0.006**)	0.268****
Procalcitonin (ng/mL) (N=20)	0.7650 (0.4175 - 2.6975) (p<0.001*)	0.59 (0.41 - 1.70) (p<0.001**)	1.20 (0.385 – 5.485) (p=0.011**)	0.648****
Erythrocyte sedimentation rate (mm/h)	73.19 ± 31.891 (p=0.312*)	68.47 ± 31.423 (p=0.916**)	77.63 ± 32.698 (p=0.212**)	0.434***
International normalized ratio (INR)	1.130 (1.000-1.250) (p<0.001*)	1.070 (1.000 - 1.200) (p=0.001**)	1.175 (1.005 – 1.365) (p<0.001**)	0.243****

Obesity

This subgroup of obese patients consists of 31 obese, hemodialyzed, and hospitalized COVID-19 patients. Of these, 11 (35.48%) died during hospitalization. The characteristics are shown in Table 2. We did not find a statistically significant correlation between patient gender and mortality in this subgroup. The average age of this subgroup was 63.1 years; considering age as a scalar variable, we tested whether the patients who died were older, and we did not discover a statistically significant correlation (p=0.261) between old age and mortality. Considering age as a dichotomous categorical variable (<65 years OR ≥65 years), we tested whether patients who died were older and did not find a statistically significant correlation between older age and mortality (Table 2). In this subgroup, statistically significant associations with mortality were seen in patients presenting with lymphopenia (p=0.033) at admission (Table 2). Among the complications that occurred during hospitalization, the correlations with mortality were strongly seen in patients having infectious complications of any kind, sepsis, thrombosis, and hemorrhage (Table 2).

**Table 2:** Characteristic of patients with obesity *Fisher’s exact test; **Shapiro-Wilk test; ***student t-test; ****Mann-Whitney U test

Parameter (N=31)	Value	Survivors	Non-survivors	P (p<0.05)*,**,***,****
Gender (Nr. (%))	15 (48.4%) F, 16 (51.6%) M	11 (55.0%) F, 9 (45.0%) M	4 (36.4%) F, 7 (63.6%) M	0.458*
Age (MV ± SD) (years)	63.10 ± 8.541 (p=0.156*)	61.80 ± 7.061 (p=0.114**)	65.45 ± 10.709 (p=0.504**)	0.261***
Age group (Nr. (%))				
<41	0 (0%)	0 (0%)	0 (0%)	0.176*
41-65	17 (54.9%)	13 (65.0%)	4 (36.4%)	-
66-80	13 (41.9%)	7 (35.0%)	6 (54.5%)	-
>80	1 (3.2%)	0 (0%)	1 (9.1%)	-
Hypertension (Nr. (%))	0 (0.0%) - 31 (100%) +	0 (0.0%) - 20 (100%) +	0 (0.0%) - 11 (100%) +	-
Atherosclerosis (Nr. (%))	20 (64.5%) - 11 (35.5%) +	15 (75.0%) - 5 (25.0%) +	5 (45.5%) - 6 (54.5%) +	*0.132
Neoplasia (Nr. (%))	27 (87.1%) - 4 (12.9%) +	19 (95.0%) - 1 (5.0%) +	8 (72.7%) - 3 (27.3%) +	*0.115
Pulmonar disease (Nr. (%))	26 (83.9%) - 5 (16.1%) +	17 (85.0%) - 3 (15.0%) +	9 (81.8%) - 2 (18.2%) +	*1.000
Diabetes (Nr. (%))	15 (48.4%) - 16 (51.6%) +	12 (60.0%) - 8 (40.0%) +	3 (27.3%) - 8 (72.7%) +	*0.135
Chronic kidney disease etiology (Nr. (%)) (N=25)				
Ischemic nephropathy	14 (51.9%) - 13 (48.1%) +	7 (38.9%)	6 (66.7%)	*0.205
Diabetic nephropathy	21 (77.8%) - 6 (22.2%) +	4 (22.2%)	2 (22.2%)	-
Tubulointerstitial nephropathy	22 (81.5%) - 5 (18.5%) +	5 (27.8%)	0 (0%)	-
Multiple myeloma	25 (92.6%) - 2 (7.4%) +	2 (11.1%)	0 (0%)	-
Other glomerular causes	26 (96.3%) - 1 (3.7%) +	0 (0%)	1 (11.1%)	-
Oxigenotherapy (Nr. (%))	25 (80.6%) - 6 (19.4%) +			
Infections (Nr. (%))	16 (51.6%) - 15 (48.4%) +	12 (60.0%) - 8 (40.0%) +	4 (36.4%) - 7 (63.6%) +	0.273*
Hemorrhagic adverse events (Nr. (%))	20 (64.5%) - 11 (35.5%) +	12 (60.0%) - 8 (40.0%) +	8 (72.7%) - 3 (27.3%) +	*0.698
Thrombosis (Nr. (%))	27 (87.1%) - 4 (12.9%) +	19 (95.0%) - 1 (5.0%) +	8 (72.7%) - 3 (27.3%) +	*0.115
Death (Nr. (%))	20 (64.5%) - 11 (35.5%) +			
Hospitalization lenght (MV ± SD) (days)	16 (14-19) (p=0.009*)			
Lymphocytes (Nr. / μL)	640.0 (390.0-960.0) (p=0.001*)	700.0 (390.0-1330.0) (p=0.022**)	510.0 (370.0 – 730.0) (p=0.741**)	0.193****
Lymphopeniae (Nr. (%))	7 (22.6%) - 24 (77.4%) +	7 (35.0%) - 13 (65.0%) +	0 (0%) - . 11 (100%) +	*0.033
Hemoglobin (g/dL)	9.93 ± 2.053 (p=0.983*)	9.96 ± 1.729 (p=0.428**)	9.89 ± 2.639 (p=0.988**)	0.935***
Albumin (g/dL) (N=28)	3.55 (3.37-3.965) (p=0.014*)	3.77 (3.39 - 4.21) (p=0.011**)	3.47 (3.31-3.60) (p=0.541**)	0.094****
Ferritin(ng/mL) (N=24)	1197 (774.90-2396.0) (p=0.003*)	1164.0 (586.40 – 2396.0) (p=0.139**)	1497.50 (866.93 – 3728.25) (p=0.034**)	0.333****
Interleukin – 6 (pg/mL) (N=16)	82.2450 (17.3325-155.40) (p<0.001*)	38.17 (13.2750-133.20) (p<0.001**)	105.10 (91.310-821.40) (p=0.001**)	0.104****
C-reactive protein (mg/dL)	77.50 (29.540-157.760) (p=0.025*)	55.3750 (29.4275-160.550) (p=0.029**)	104.40 (52.540 – 157.760) (p=0.448**)	0.433****
Procalcitonin (ng/mL) (N=20)	0.690 (0.40-2.690) (p<0.001*)	0.60 (0.390 - 3.210) (p<0.001**)	0.890 (0.3775 – 2.60) (p=0.010**)	0.846****
Erythrocyte sedimentation rate (mm/h)	89 (59.50-110.25) (p=0.042*)	85.32 ± 26.675 (p=0.271**)	81.73 ± 38.3510 (p=0.168**)	0.765***
International normalized ratio (INR)	1.060 (1.0 - 1.20) (p<0.001*)	1.065 (1.005 – 1.200) (p<0.001**)	1.060 (0.990 - 1.220) (p=0.393**)	0.951****

Diabetes mellitus

In this subgroup, 19 patients (70.4%) are male and eight patients (29.6%) are female (Table 3). We did not find a statistically significant correlation (p=0.405) between patient gender and mortality in this subgroup. Interleukin-6 was found to be statistically significant associated with mortality (Table 3).

**Table 3:** Characteristics of patients with diabetes *Fisher’s exact test; **Shapiro-Wilk test; ***student t-test; ****Mann-Whitney U test

Parameter (N=31)	Value	Survivors	Non-survivors	p (p<0.05)*,**,***,****
Gender (Nr. (%))	8 (29.6%) F, 19 (70.4%) M	6 (37.5%) F, 10 (62.5%) M	2 (18.2%) F, 9 (81.8%) M	0.405*
Age (MV ± SD) (years)	65.11 ± 12.44 (p = 0.231*)	62.13 ± 11.977 (p=0.276**)	69.45 ± 12.356 (p=0.067**)	0.135***
Age group (Nr. (%))				
<41	1 (3.7%)	1 (6.3%)	0 (0%)	0.246*
41-65	11 (40.7%)	8 (50.0%)	3 (27.3%)	-
66-80	13 (48.1%)	7 (43.7%)	6 (54.5%)	-
>80	2 (7.4%)	0 (0%)	2 (18.2%)	-
Obesity (Nr. (%)) (N=24)	7 (30.4%) - 16 (69.6%) +	6 (42.9%) - 8 (57.1%) +	1 (11.1%) - 8 (88.9%) +	0.176*
Hypertension (Nr. (%))	0 (0%) - 27 (100%) +	0 (%) - 16 (100%) +	0 (0%) - 11 (100%) +	-
Atherosclerosis (Nr. (%))	14 (51.9%) - 13 (48.1%) +	8 (50%) - 8 (50%) +	6 (54.5%) - 5 (45.5%) +	1.000*
Neoplasia (Nr. (%))	25 (92.6%) - 2 (7.4%) +	16 (100%) - 0 (%) +	9 (81.8%) - 2 (18.2%) +	0.157*
Pulmonar disease (Nr. (%))	23 (85.2%) - 4 (14.8%) +	14 (87.5%) - 2 (12.5%) +	9 (81.8%) - 2 (18.2%) +	1.000*
Chronic kidney disease etiology (Nr. (%)) (N=25)				
Ischemic nephropathy	3 (12.5%) - 21 (87.5%) +	13 (86.6%)	8 (88.9%)	1.000*
Diabetic nephropathy	22 (91.7%) - 2 (8.3%) +	1 (6.7%)	1 (11.1%)	-
Other glomerular causes	23 (95.8%) - 1 (4.2%) +	1 (6.7%)	0 (0%)	-
Oxigenotherapy (Nr. (%))	20 (74.1%) - 7 (25.9%) +			
Infections (Nr. (%))	16 (59.3%) - 11 (40.7%) +	14 (87.5%) - 2 (12.5%) +	2 (18.2%) - 9 (81.8%) +	0.001*
Hemorrhagic adverse events (Nr. (%))	21 (77.8%) - 6 (22.2%) +	12 (75%) - 4 (25%) +	9 (81.8%) - 2 (18.2%) +	1.000*
Thrombosis (Nr. (%))	25 (92.6%) - 2 (7.4%) +	16 (64.0%) - 9 (36.0%) +	0 (0.0%) - 2 (100%) +	*0.157
Death (Nr. (%))	16 (59.3%) - 11 (40.7%) +			
Hospitalization lenght (MV ± SD) (days)	16 (13-20) (p=0.041*)	17.31 ± 7.643 (p=0.071**)	16.18 ± 4.792 (p=0.589**)	0.662***
Lymphocytes (Nr. / μL)	720 (470-1500) (p=0.014*)	955.0 (497.5-1655.0) (p=0.213**)	620.0 (470.0-850.0) (p=0.002**)	0.267****
Lymphopeniae (Nr. (%))	10 (37%) - 17 (63%) +	8 (50.0%) - 8 (50.0%) +	2 (18.2%) - 9 (81.8%) +	0.124*
Hemoglobin (g/dL)	10.52 ± 1.851 (p=0.952*)	10.36 ± 1.597 (p=0.990**)	10.75 ± 2.232 (p=0.984**)	0.593***
Albumin (g/dL) (N=28)	3.0672 ± 0.56935 (p=0.254*)	3.940 (3.420-4.020) (p=0.010**)	3.465 (3.310-3.8275) (p=0.090**)	0.346****
Ferritin(ng/mL) (N=24)	1143.00 (662.85-1952.50) (p<0.001*)	831.0 (512.60-1506.50) (p=0.106**)	1598.0 (866.93-2583.75) (p=0.025**)	0.082****
Interleukin – 6 (pg/mL) (N=16)	73.18 (27.01-153.05) (p<0.001*)	44.590 (12.560-73.0425) (p=0.068**)	144.90 (94.620-1450.20) (p=0.037**)	0.013****
C-reactive protein (mg/dL)	59.04 (20.45-146.76) (p=0.017*)	44.31 (18.18-131.155) (p=0.025**)	116 (52.54-186.24) (p=0.693**)	0.114****
Procalcitonin (ng/mL) (N=20)	0.5 (0.36-1.903) (p<0.001*)	0.43 (0.36-0.79) (p<0.001**)	1.2 (0.41-2.87) (p=0.154**)	0.239****
Erythrocyte sedimentation rate (mm/h)	86.52 ± 23.173 (p=0.356*)	85.93 ± 22.595 (p=0.531**)	87.27 ± 24.98 (p=0.669**)	0.889***
International normalized ratio (INR)	1.085 ± 0.1124 (p=0.246*)	1.068 ± 0.092 (p=0.920**)	1.11 ± -0.1377 (p=0.479**)	0.344***

## Discussion

During the COVID-19 pandemic, CKD was an important risk factor for morbidity and mortality. The study that we have presented demonstrates that for the subgroup of hemodialyzed patients diagnosed with coronary artery disease and hospitalized for COVID-19, older age is a risk factor for mortality. A series of medical studies found in the current literature share similar results with the presented study. Thus, the study presented by Loffi et al., which included patients with coronary artery disease and COVID-19, showed that advanced age is a risk factor for mortality (p<0.001). CKD was not found to be a risk factor for mortality (p=0.134) [16]. However, Ciceri et al. highlight risk factors for mortality in hospitalized patients with COVID-19, both advanced age and the presence of some comorbidities, including CKD and ischemic heart disease, as well as an increased number of concurrent comorbidities in patients included in the study [17].

The present study found that infectious complications of any kind are correlated with mortality in hemodialyzed patients with coronary artery disease hospitalized for COVID-19. Looking at the odds of patients hospitalized for COVID-19 developing sepsis, Kurt et al. showed that neither CKD nor coronary artery disease are risk factors for the development of septicemia in COVID-19 patients [18]. Together, the current results and those of Kurt et al. suggest that patients with CKD and coronary artery disease hospitalized for COVID-19 are not at increased risk of developing sepsis, but when at risk of sepsis, the negative impact on survival is pronounced. Regarding the subgroup of obese patients hospitalized for COVID-19, the present study found lymphopenia as a risk factor for mortality. This information follows Foulkes et al.'s finding that there is a causal link between obesity and death from COVID-19 involving leukocyte levels [19]. Thus, the question arises whether CKD may also be involved in this causal link. Certainly, yes, given the impact of adipose tissue in the development of CKD [20]. Lymphopenia is associated in the current study with mortality in obese patients with CKD hospitalized for COVID-19. In patients hospitalized for COVID-19, Birtay et al. discovered that low serum lymphocyte levels are associated with death [21]. We believe that a future direction of study is the influence of CKD and obesity on mortality in COVID-19 patients, with increased emphasis on lymphopenia as a marker of this association. Infectious complications are a risk factor for death in COVID-19 patients. Gasmi et al. show that the increased mortality of obese COVID-19 patients may have an etiopathogenic element of an immune nature [22]. Immune involvement in increased mortality in patients with COVID-19 and obesity may suggest why the link between obesity, altered immunity, and sepsis is particularly detrimental in the presence of another comorbidity that alters proinflammatory immune status, namely CKD [23,24]. For the subgroup of diabetic patients hospitalized for COVID-19, the risk factors for death are: increased serum level of IL-6 and the development of infectious complications of any kind. Luo et al. confirm that the increased level of IL-6 is a risk factor for death in patients with COVID-19, diabetes, and other comorbidities, including CKD [25]. Future research may elucidate whether the development of infectious complications and sepsis contribute significantly to the mortality of these patients or not.

This study has potential limitations. One of them refers to a small number of patients. Another identified limitation is the retrospective nature of the study.

## Conclusions

Older age was an important risk factor for mortality and morbidity in COVID-19 hospitalized patients, unconcerned with patients associated comorbidities. Obesity was associated with increased mortality from infectious causes in patients with advanced CKD. We found an important inflammatory process that accompanies advanced chronic renal damage, which could explain the severe impact of infections on the increased morbidity and mortality in this category of patients. Interleukin-6 proved to have an important role in the pathophysiology of SARS-COV2 infection and probably also in other infections, a role insufficiently studied, especially in CKD patients. We state an additional argument for exploring the pathophysiological role of IL-6 as well as the therapeutic role of IL-6 blockers. It is certain that the COVID-19 pandemic has brought some lessons for medical professionals. As new waves of COVID-19 emerge, mitigation strategies to reduce the risk of exposure of high-risk populations to the virus remain of primary concern.
